# Transcriptome Analysis of Honeybee (*Apis Mellifera*) Haploid and Diploid Embryos Reveals Early Zygotic Transcription during Cleavage

**DOI:** 10.1371/journal.pone.0146447

**Published:** 2016-01-11

**Authors:** Camilla Valente Pires, Flávia Cristina de Paula Freitas, Alexandre S. Cristino, Peter K. Dearden, Zilá Luz Paulino Simões

**Affiliations:** 1 Departamento de Genética, Faculdade de Medicina de Ribeirão Preto, Universidade de São Paulo, Ribeirão Preto, Brazil; 2 The University of Queensland, Diamantina Institute, Translational Research Institute, Brisbane, Australia; 3 Genetics Otago and Gravida, the National Centre for Growth and Development, Biochemistry Department, University of Otago, Dunedin, New Zealand; 4 Departamento de Biologia, Faculdade de Filosofia, Ciências e Letras de Ribeirão Preto, Universidade de São Paulo, Ribeirão Preto, Brazil; University of Illinois, UNITED STATES

## Abstract

In honeybees, the haplodiploid sex determination system promotes a unique embryogenesis process wherein females develop from fertilized eggs and males develop from unfertilized eggs. However, the developmental strategies of honeybees during early embryogenesis are virtually unknown. Similar to most animals, the honeybee oocytes are supplied with proteins and regulatory elements that support early embryogenesis. As the embryo develops, the zygotic genome is activated and zygotic products gradually replace the preloaded maternal material. The analysis of small RNA and mRNA libraries of mature oocytes and embryos originated from fertilized and unfertilized eggs has allowed us to explore the gene expression dynamics in the first steps of development and during the maternal-to-zygotic transition (MZT). We localized a short sequence motif identified as TAGteam motif and hypothesized to play a similar role in honeybees as in fruit flies, which includes the timing of early zygotic expression (MZT), a function sustained by the presence of the *zelda* ortholog, which is the main regulator of genome activation. Predicted microRNA (miRNA)-target interactions indicated that there were specific regulators of haploid and diploid embryonic development and an overlap of maternal and zygotic gene expression during the early steps of embryogenesis. Although a number of functions are highly conserved during the early steps of honeybee embryogenesis, the results showed that zygotic genome activation occurs earlier in honeybees than in *Drosophila* based on the presence of three primary miRNAs (pri-miRNAs) (ame-*mir-375*, ame-*mir-34* and ame-*mir-263b*) during the cleavage stage in haploid and diploid embryonic development.

## Introduction

Embryonic development is the result of a precisely controlled sequence of events modulated by environmental and intracellular signals [1]. Early embryogenesis is characterized by maternal elements that are preloaded into eggs and act directly on egg activation, cleavage, and zygotic genome activation (ZGA) [2]. The zygotic products gradually replace these maternal regulatory elements and pathways. In Hymenoptera, this process deserves special attention because two developmental pathways occur, wherein fertilized eggs develop as females (diploid) and unfertilized eggs develop as males (haploid), thus leading to a sex determination system termed haplodiploidy. However, the developmental strategies of honeybees during early embryogenesis are still unknown.

At the beginning of embryogenesis, maternal RNA families [3] are recruited for translation [4] because the RNA molecules required for egg activation are not transcribed in the oocyte or in early embryos [3]. The mRNAs that encode proteins remain inactive from the time the oocyte matures[5] but have different fates and timing during development [6]. The zygotic genome remains quiescent during the initial developmental events [1,7] and limited to no transcription occurs. The translation of maternal mRNAs are translated to proteins in a time dependent manner, and these proteins regulate the expression of zygotic genes [8] during the maternal-to-zygotic transition (MZT) [2]. The MZT is a dynamic process whereby the degradation of maternal protein and RNA occurs simultaneously with transcriptional reactivation for the synthesis of new molecules, thus ensuring the essential events of embryonic development [2]. Two waves of transcription (minor and major) characterize the activation of the zygotic genome [9–11], and these events are common to different organisms, such as mice, frogs, fish, fruit flies, sea urchins and nematodes [2,12]. In *Drosophila*, the minor wave starts during the 8^th^ mitotic cycle in the pre-blastoderm embryo, and the major wave occurs at the 14^th^ mitotic cycle in the cellular blastoderm [9,11,13,14]. In general, the mRNAs expressed during the first wave are involved in sex determination, patterning, and cellularization [13]. Transcription factors such as Zelda (*vielfaltig-vlf*) are involved in the two waves of ZGA by binding to *cis*-element conserved TAGteam motifs [13,15,16]. In *Drosophila*, *zelda* is described as a maternal transcript that is degraded in early embryogenesis, although it is produced by zygotic machinery during blastoderm cellularization [15] when the number of transcribed mRNAs is high as demonstrated through genome-wide approaches [1,17–19]. In honeybees, the time required to achieve the 8^th^ or 14^th^ mitotic cycles is unknown. Cleavage has been suggested to occur 7 h after egg laying (AEL), and blastoderm formation has been suggested to occur approximately 18–24 h AEL [20,21]. In honeybees, studies during embryogenesis have generated important data on anterior-posterior (AP) and dorsal-ventral (DV) axis determination [22–32], although studies during early embryogenesis are lacking. Therefore, information on the dynamics of gene expression and the pathways and timing of honeybee development could provide insight into the early development of both types of embryos.

Most of the studies of haplodiploid development have focused on sex and caste determination processes and are limited to the larvae, pupae or adult stages of honeybees [33] and wasps (*Nasonia sp*.) [34,35]. Our understanding of the early events of embryogenesis in insects is primarily derived from *Drosophila melanogaster*, a diploid organism with non-conserved processes of early sex determination by dosage compensation [36]. Haploid early developmental events, such as translation [17,37], cytoplasmic polyadenylation [37,38], RNA interference activation [39,40], phosphorylation [5] and maternal RNA degradation [1,41–43] have already been described in haploid embryos of *Drosophila* using artificially activated eggs. Therefore, honeybees constitute the ideal model for studying haploid development because haplodiploidy occurs naturally in these organisms.

In this study, we have addressed questions raised by the differential development of males (haploid) and females (diploid) of *Apis mellifera* (*A*. *mellifera*), with special reference to egg activation and the MZT. We constructed and analyzed fourteen RNA-seq libraries of mature oocytes and haploid and diploid development at different ages (2 h, 6 h and 18–24 h of development). The dynamics of gene expression in both types of embryos and predicted microRNA (miRNA)-target interactions demonstrated conserved mRNAs and biological processes between the two types of embryos and between *D*. *melanogaster* and *A*. *mellifera*. *In situ* hybridization and gene expression analyses related to the MZT have confirmed the conserved features of the embryogenesis process among insects, particularly among the aforementioned organisms. Finally, the early expression of ame-*mir-375-3p*, ame-*mir-34-5p* and ame-*mir-263b-5p* prior to cellular blastoderm formation demonstrated the early activation of zygotic transcription in honeybees.

## Material and Methods

### Sample collection and RNA-seq library preparation

All of the samples were obtained from honeybee colonies maintained in the apiary of the Department of Genetics at the University of São Paulo, Ribeirão Preto, Brazil. To sample age-controlled embryos, a mated egg-laying queen was confined on a wax comb frame in an egg- and brood-free area and placed back into the hive. After 2 h, the eggs were collected and pooled as a 0–2 h diploid embryo sample. Six hours later, the cage was removed and the queen was released, and a 0–6 h diploid embryo sample was collected. The remaining embryos were collected 24 h later as the 18–24 h diploid embryo sample. A virgin queen was caged within a male (drone) comb frame to obtain haploid embryos with the same timings. Mature oocytes were collected directly from the queen ovaries. We collected 50–100 oocytes or embryos per sample.

**Fig 1:**
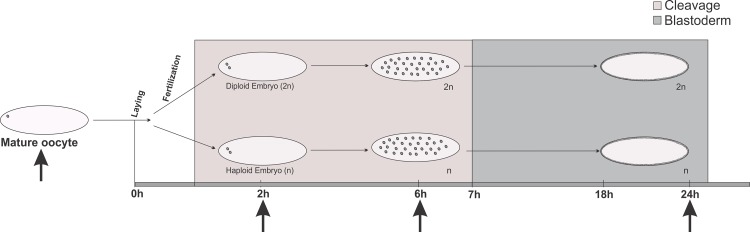
*Apis mellifera* developmental phases used for sample collection and RNA sequencing. Mature oocytes were collected directly from queen ovaries and age-controlled haploid and diploid embryos (at 0–2, 0–6 and 18–24 h after egg laying) from queenright or queenless *Apis mellifera* colonies (arrows).

Total RNA was extracted from oocytes and embryos using TRIzol® (Life Technologies), and the concentration and purity of the samples were determined using a NanoDrop spectrometer (Thermo Scientific). Four micrograms of total RNA was used as a template for the small RNA and mRNA libraries, which were prepared according to the Illumina single-end protocol (Genome Analyzer II, Life Sciences). The library preparation and sequencing were performed at the High-Throughput Sequencing Facility of the University of North Carolina, Chapel Hill, North Carolina, USA (http://www.unc.edu/htsf/index.html). A total of fourteen libraries were constructed using age-controlled haploid and diploid embryos as well as mature oocytes. Seven mRNA and seven small RNA libraries were prepared from mature oocytes (reads with 50 bp), diploid embryos at 0–2 h (50 bp reads), 0–6 h (36 bp reads) and 18–24 h (36 bp reads) AEL, and haploid embryos at 0–2 h (50 bp reads), 0–6 h (36 bp reads) and 18–24 h (50 bp reads) AEL (Fig 1).

The libraries were deposited in the Sequence Read Archive database (SRA) at the NCBI (National Center for Biotechnology Information), and the accession numbers are as follows: mature oocytes, mRNA SRR1539287 and small RNA SRR1539288; haploid embryos at 0–2 h, mRNA SRR1538449 and small RNA SRR1538718; haploid embryos at 0–6 h, mRNA SRR1538849 and small RNA SRR1538749; haploid embryos at 0–24 h, mRNA SRR1538850 and small RNA SRR1538876; diploid embryos at 0–2 h, mRNA SRR1538934 and small RNA SRR1538974; diploid embryos at 0–6 h, mRNA SRR1538877 and small RNA SRR1538878; and diploid embryos at 0–24 h, mRNA SRR1539284 and small RNA SRR1539264.

### Bioinformatics analysis of RNA-seq data

Adaptor sequences were filtered out from both the mRNA and small RNA libraries using Cutadapt [44]. The Cutadapt tool was used to trim the size of reads to 36 nt in all libraries. The sequenced reads were mapped using BWA software [45] version 0.7.0 and default parameters. To identify the genetic elements that compose the mRNA and small RNA libraries, the reads were mapped to a database containing honeybee predicted genes (Amel4.5; http://genome.ucsc.edu), tRNA (GtRNAdb; http://gtrnadb.ucsc.edu/), rRNA (SILVA; http://www.arb-silva.de/), pathogen mRNAs (http://www.ncbi.nlm.nih.gov/), other noncoding RNA (NONCODE; http://www.noncode.org/NONCODERv3/) and miRNA (miRBase 19; http://www.mirbase.org/). The 19–24 nt long reads from the small RNA libraries were also mapped into the miRNA hairpin sequences (miRBase version 19) [46]. According to the position of the reads mapped into the hairpins, they were classified as 'miRNA-5p' (5-prime end, reads mapped in 5’-end of hairpin, arm-5p) or 'miRNA-3p' (3-prime end; reads mapped in 5’-end of hairpin, arm-3p). Expression data were assessed by counting the number of reads mapped to each predicted honeybee gene (mRNA) or miRNA. Subsequently, the read counts were normalized in RPKM (reads per kilobase per million mapped reads) according to Mortazavi *et al*. [47], and our cut-off for RPKM normalization was 5 mapped reads. We considered only the uniquely mapped reads to identify protein coding genes (mRNAs) and miRNAs expressed in our libraries and expression levels according to Mortazavi et al [47].

### Characterizing classes of genes (mRNA and miRNA) according to expression profiles

We classified the mRNAs and miRNA into three classes according to their expression profiles. The expression values (RPKM) of the mRNAs and miRNA identified in the mature oocyte and haploid and diploid embryos at 0–2 h, 0–6 h and 18–24 h were analyzed using the DEGseq package [48] version 1.16.0 to identify differentially expressed mRNAs and miRNA (p<0.05). The DEGseq method was used to determine the significant fold changes of transcripts among different time ranges. Among all of the available models for DEGseq, MARS (MA-plot-based method with random sampling model) was used as the primary model for DEGseq.

We compared the mature oocytes with 0–2 h embryos, 0–2 h embryos with 0–6 h embryos, mature oocytes with 0–6 h embryos, and 0–6 h embryos with 18–24 h embryos. Based on the results, we classified the expressed mRNAs and miRNAs into three different classes: class I, mRNAs that were expressed in mature oocytes but decreased in expression continually until 18–24 h (increased expression in mature oocytes compared with that of the “mature oocytes vs 0–2 h embryos” and/or “mature oocytes vs 0–6 h embryos" and increased expression of 0–6 h embryos compared with “0–6 h embryos vs 18–24 h embryos" at p<0.5); class II, mRNAs and miRNA that were strictly zygotic (with no transcripts in mature oocytes but noticeable expression in embryos at 0–2 h, 0–6 h and 18–24 h AEL) and mRNAs and miRNA whose expression increased as embryogenesis progressed (increased expression in 0–2 h and/or 0–6 h embryos compared with “mature oocytes vs 0–2 h embryos” and/or “mature oocytes vs 0–6 h embryos" and increased expression at 18–24 h compared with “0–6 h embryos vs 18–24 h embryos" at p<0.5); and class III, mRNAs and miRNA that were expressed in mature oocytes whose expression decreased (or was absent) during cleavage (0–2 h and 0–6 h) and then increased again at 18–24 h (increased expression in mature oocytes compared with “mature oocytes vs 0–2 h embryos” and “mature oocytes vs 0–6 h embryos" and increased expression at 18–24 h compared with “0–6 h embryos vs mature oocytes" at p<0.5).

### Functional annotation analysis

The honeybee and fruit fly orthologous genes were identified by reciprocal best hits using BLAST (Basic Local Alignment Search Tool) [49]. To explore the functional role played by zygotic and maternal genes, we searched for biological processes as described in the Gene Ontology (GO) database using DAVID 6.7 [50] and default parameters.

### Identification of the TAGteam motif in the promoter region of zygotic mRNAs

We searched for conserved motifs in the promoter region of the zygotic mRNAs using SCOPE (Suite for Computational Identification of Promoter Elements), which is a parameter-free program designed to identify *cis* sequences in a set of co-regulated mRNAs (http://genie.dartmouth.edu/scope/) [51].

The search for motifs was performed in a 1000 bp region upstream of the start codon of the class II mRNAs (haploid and diploid). We selected the motifs based on their coverage (greater than 80%) and similarity to TAGteam motifs (ten Bosch *et al*. 2006) and AGGTA-containing (or its reverse complement TACCT) sequences. The position weight matrices (PWMs) generated for each motif identified by SCOPE were aligned to the TAGteam motif PWMs from *D*. *melanogaster* and *A*. *aegypti* according to Biedler *et al*. [18]. The motifs identified for the zygotic mRNAs were also aligned to TAGteam motif sequences [13], CAGGTAG, CAGGCAG and TAGGTAG, using the STAMP program (http://www.benoslab.pitt.edu/stamp/). STAMP was also used to search the identified motifs for zinc-finger domains, which is a feature displayed by the TAGteam motif bound by Zelda (ten Bosch *et al*. 2006).

### Predicted targets for miRNAs

The predicted miRNA targets were determined as described by Dong *et al*. [52] and Zhang *et al*. [53]. First, a Pearson correlation coefficient was calculated for every possible miRNA-target pair using the expression profiles provided by the RNA-seq of the mature oocytes and haploid and diploid embryos of 0–2 h, 0–6 h, 18–24 h embryos. We identified negatively correlated miRNA-target pairs (R<-0.8) for both haploid and diploid embryos separately and searched for miRNA binding sites at the 3’ untranslated region (3’UTR) of the negatively correlated genes (mRNA) using miRanda software [54]. Because the 3’UTR data are incomplete for the honeybee genome, we used an arbitrary 1000 bp region downstream of the stop codon for each predicted gene. The predicted miRNA-target pairs with a minimum free energy of <-15 kcal/mol were considered for putative miRNA-target interactions. Cytoscape (http://cytoscape.org) was used as a network graphic to visualize the predicted miRNA-target interactions. Only orthologous genes were considered to be shown in the network graphic. This information allowed us to infer the functionality according to what was described for *Drosophila*.

### Localization of mRNA and pri-miRNA by *in situ* hybridization

*In situ* hybridization probes were synthesized using embryonic cDNA (complementary DNA) as a template. Regions varying from 300 to 700 bp were amplified from mRNA and primary miRNA (pri-miRNA) using specific primers, cloned into the appropriated vector, and then sequenced. The cloned fragments were used to produce the RNA sense and antisense *in situ* hybridization probes. The following primer sequences were used: ame-*zelda* F 5' GTACTACGATGCCTCACCAG 3' and R 5' TGATCATACCGTCCGTGTTC 3', pri-ame-*mir-263b* F 5' TCTCTCCACGACTGCAAGAA 3' and R 5' ATTGTTGATCAAAGGATTCCTCA 3', pri-ame-*mir-34* F 5' TCATCGAATTCACCGTGGAG 3' and R 5' TAATCACCGGGAGAATACTGA 3', pri-ame-*mir-375*, F 5' GTACTTGTTCGCGCGTTCTC 3' and R 5' CGCTGCATATACACCAACTGA 3'. The protocol used to localize *zelda* transcripts was performed according to Osborne and Dearden [55], and the pri-miRNA *in situ* hybridizations were performed according to Zondag *et al*. [29]. The negative controls (sense probes) were also made according to Osborne and Dearden [55]. The images were visualized and captured using an Olympus BX61 microscope with a DP71 camera.

### Identification of pri-miRNA by polymerase chain reaction (PCR)

Triplicate RNA samples from the mature oocytes and haploid and diploid embryos at 2 h and 6 h were incubated in the presence of three units of RNase-free DNase (Invitrogen) for 15 min at room temperature and for 5 min at 70°C to inactivate the DNase. First-strand cDNA was synthesized by reverse transcription (RT) using 2.5 μg total RNA, SuperScript II reverse transcriptase and the oligo (dT) 12–18 primer (Invitrogen, Life Technologies). Reactions that did not include the SuperScript II reverse transcriptase or cDNA template were prepared as negative controls and analyzed in parallel. PCR amplification was conducted using 10 μL Master Mix 2X (Promega), 7.4 μL water, 0.8 μL each gene-specific primer (10 mM) and 1 μL cDNA. The PCR conditions were 95°C for 30 s, 60°C for 45 s and 72°C for 30 s. The PCR product was analyzed using electrophoresis on a 2.0% agarose gel (Amersham-Pharmacia Biotech) in TBE 1x (89 mM Tris, 89 mM boric acid, 2 mM EDTA, pH 8.0), with 0.5 μg ethidium bromide per mL. The following primer sequences were used: pri-ame-*mir-263b* F 5' TCGGTATCCAGCAAGTGATG' and R 5' GTGGAGGAAAACGAGGAGAA 3'; pri-ame-*mir-34* F 5' CCCTACGATCGTCATAATTG 3' and R 5' GGAGGGAATGCTTGACGAAA 3'; pri-ame-*mir-375*, F 5' CATCCATTCAGTTTGATAACTC 3' and R 5' ATCGATTGAATTATCAGTTTGG 3'.

## Results

### Sequencing libraries description

**Fig 2:**
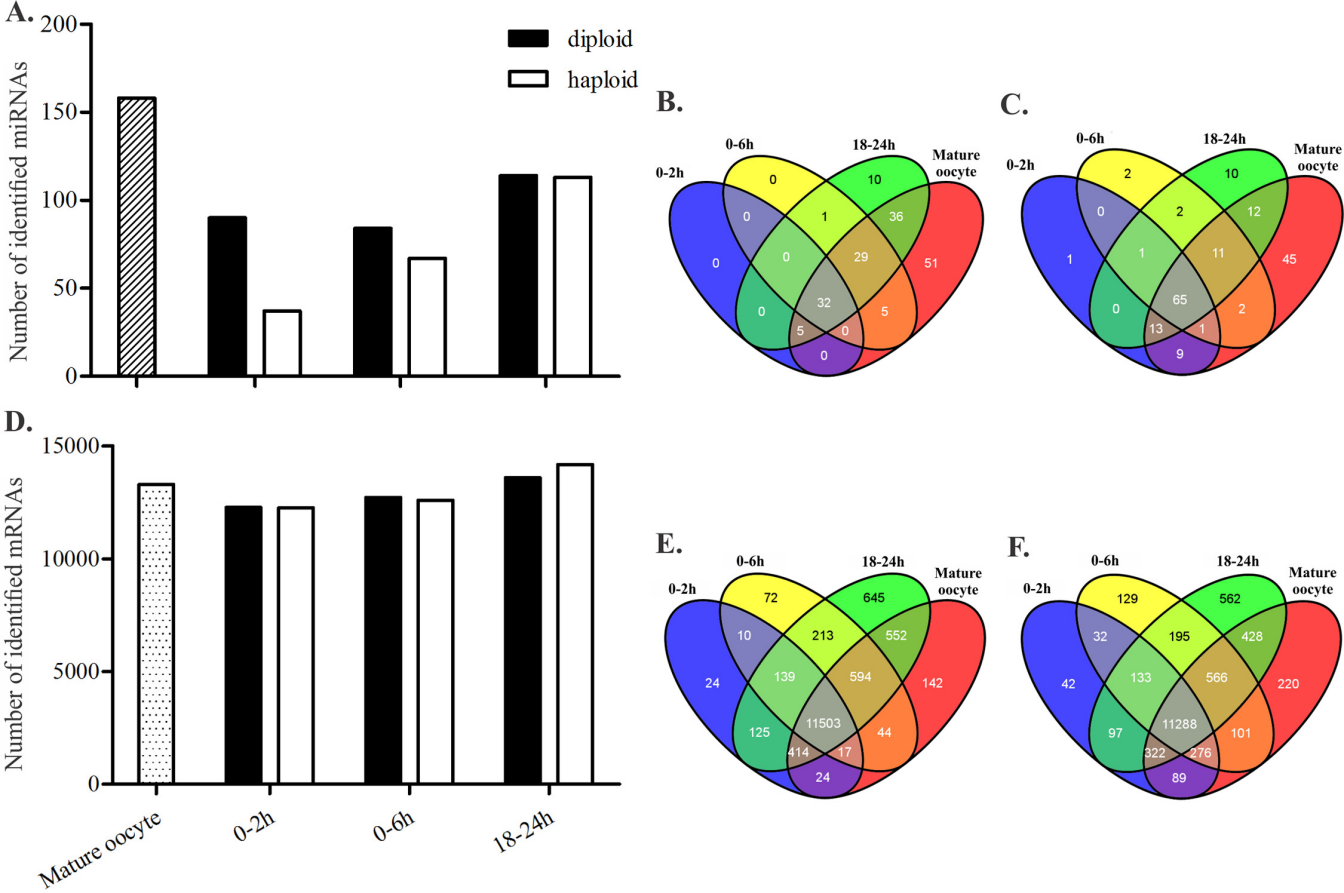
Total number of identified miRNAs and mRNAs in each library of haploid and diploid embryos, and mature oocytes of *Apis mellifera*. The total number of miRNAs (A) and mRNAs (D) identified in each library. Venn diagrams show the number of miRNAs (B and C) and mRNAs (E and F) shared or exclusively expressed in each analyzed developmental stage of haploid (B and E) and diploid (C and F) embryos.

Seven small RNA libraries and seven mRNA libraries generated from the mature oocytes, haploid embryos, and diploid embryos (Fig 1) were mapped to a database of honeybee transcripts as described in the Materials and Methods section. More than 90% of the mRNA reads were mapped to the annotated *A*. *mellifera* coding genes (Official Gene Set 3.2) (see Table 1). The 19–24 nt long reads from small RNA libraries were mapped to precursor miRNA sequences (S1 Fig). The 19–24 nt long reads from small RNA libraries were mapped to precursor miRNA sequences (S1 Fig). A higher amount of miRNA and mRNA was found in the mature oocytes compared with that of the early embryos (0–2 h and 0–6 h) (Fig 2). The number of expressed miRNA and mRNA increased during the blastoderm stage (18–24 h) (Fig 2). In general, the diploid embryos presented a greater diversity of miRNA during embryogenesis compared with the haploid embryos. However, the amount of mRNA expressed in both the haploid and diploid embryos was approximately the same (Fig 2).

**Table 1:** *Apis mellifera* mRNA and small RNA composition in each sequenced sample. Mature oocytes and 0–2 h, 0–6 h and 18–24 h haploid and diploid embryos were submitted to RNA-seq, and the number of reads generated (Raw data), mapped to the *A*. *mellifera* transcript database as described in the Material and Methods section.

			Number of reads							
			Total (Raw data)	mapped	microRNA	Annoted genes (*A*. *mellifera*)	Noncode	rRNA	tRNA	Pathogens
**mRNA libraries**	**Mature Oocyte**		82208246	47939930	291	47780988	120439	7385	30	30797
	**Diploid Embryos**	**0-2h**	22902101	15502664	84	15492088	7622	334	2	2534
		**0-6h**	40486094	26661385	177	26619330	35564	598	6	5710
		**0-24h**	33295431	20751326	431	20631201	101424	1219	9	17042
	**Haploid Embryos**	**0-2h**	17417565	11526057	44	11515606	7407	439	3	2558
		**0-6h**	37086884	23655878	188	23623252	28863	374	8	3193
		**0-24h**	163202336	87698424	1812	87258581	308725	39799	65	89442
**Small RNA Libraries**	**Mature Oocyte**		32955026	898309	6256	1995689	928113	14165	1199	251353
	**Diploid Embryos**	**0-2h**	17060175	1249412	6879	176645	97534	442	87	5689
		**0-6h**	37487408	287276	6386	230815	65112	323	189	11023
		**0-24h**	40213259	333157	25588	235739	54577	314	394	16545
	**Haploid Embryos**	**0-2h**	9899631	10921	171	7245	3273	14	0	218
		**0-6h**	38030892	118550	1611	88706	23682	120	122	4309
		**0-24h**	32219748	733618	27115	500389	191466	798	115	13735

Based on the *Drosophila* annotated genes (http://flybase.org/), the orthologous of sequenced mRNAs were identified and used to determine the molecular relationships driving early embryonic developmental processes in honeybees (S2 Table). In cleavage stage of honeybee embryos transcripts of *eve* (*even skipped*/GB49029) and *run* (*runt*/GB52719) [22] were localized; and during blastoderm formation, *eve* [22,26] and ame-*miR-184* [29] were found in libraries of haploid and diploid embryos at 0–2 h, 0–6 h, and 18–24 h (S1 Table). The embryonic timetable used for sample collection was based on the classic works of Nelson (1915) and DuPraw (1967) (S1 Table). Our results showed that the intervals for cleavage, 0–2 h and 0–6 h, and for blastoderm formation, 18–24 h, were adequate for the characterization of these phases of development.

### Gene expression profiles in the early embryonic development of *A*. *mellifera*

**Fig 3:**
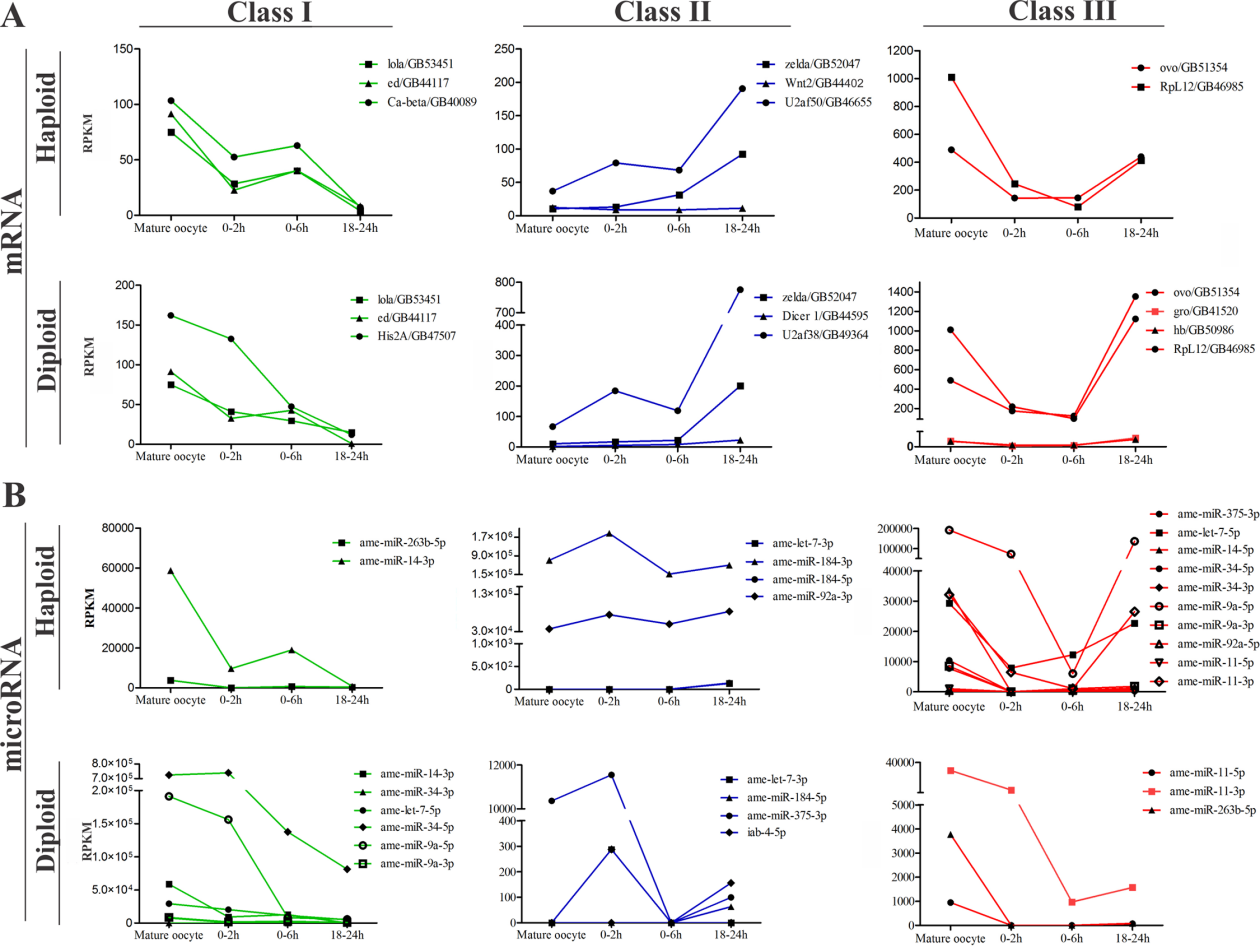
Expression profile of certain mRNAs and miRNA of each class in the early embryogenesis of *Apis mellifera*. (A) Profile of mRNAs belonging to the three different classes of genes: I, II, and III. Examples of mRNAs from class I, II and III are plotted in a separated graphic for the haploid and diploid embryos. (B) Profile of miRNAs belonging to class I, II, and III. Note the differential expression and arm use among the embryo types (such as ame-*miR-184-3p* and *5p* of class II and ame-*miR-263-5p* of class II in haploids but class III in diploids). The total number of identified mRNAs for each class is as follows: class I, 667 in diploid and 475 in haploid embryos; class II, 2680 in diploid and 475 in haploid embryos; class III, 263 in diploid and 147 in haploid embryos. The total number of miRNAs for each class is as follows: class I, 90 in diploid and 72 in haploid embryos; class II, 23 in diploid and 24 in haploid embryos; class III, 37 in diploid and 67 in haploid embryos. Overall, the distribution demonstrates specific gene expression dynamics for mRNAs and miRNA in both types of embryos. The Expression and DEGseq output of the mRNAs and miRNAs plotted here are listed in S5 Table.

We found 13,290 protein coding mRNA expressed in the mature oocytes, and 14,260 in diploid embryos and 14,376 in haploid embryos at 0–2 h, 0–6 h and 18–24 h of development. In the same periods of development, 158 miRNAs were expressed in mature oocytes, 288 in diploid embryos and 217 in haploid embryos (Fig 2). The majority of miRNAs expressed in honeybee embryogenesis were found to be highly expressed in mature oocytes. The expression profiles of the mRNA and miRNAs during the MZT process led to the classification of these transcripts into three different classes (I, II and III) according to their profiles in development (Fig 3, S3 Table and S4 Table) as described by Benoit *et al*. [17], Thomsen *et al*. [1] and Siddiqui *et al*. [19]. Class I included mRNAs and miRNAs only detected in mature oocytes or transcripts expressed in mature oocytes that had decreased expression until 18–24 h. Class I appeared to be maternal transcripts that were undergoing degradation. Class II included mRNAs and miRNAs that were undetected in mature oocytes but had significant expression in the 0–2 h, 0–6 h and 18–24 h embryos and mRNAs and miRNAs with increased expression as embryogenesis progressed as evidence of zygotic transcription. Class III corresponded to mRNAs and miRNAs expressed in mature oocytes with decreased (or absent) expression during cleavage (0–2 h and 0–6 h) and increased expression in the 18–24 h embryos. Class III mRNAs encoded transcripts that were rapidly degraded during cleavage and then replaced by the zygotic transcripts during the blastoderm stage (18–24 h). Certain transcripts (both mRNAs and miRNA) that were not expressed in a pattern consistent with any of these profiles were considered unclassified. The majority of the mRNAs were classified into class II in both the haploid (2937 mRNAs) and diploid (2680 mRNAs) embryos. The miRNAs expressed in both the haploid and diploid were enriched by class I transcripts (72 and 90 miRNAs, respectively) (S5 Table). The expression profile pattern of each class is illustrated in Fig 3.

**Fig 4:**
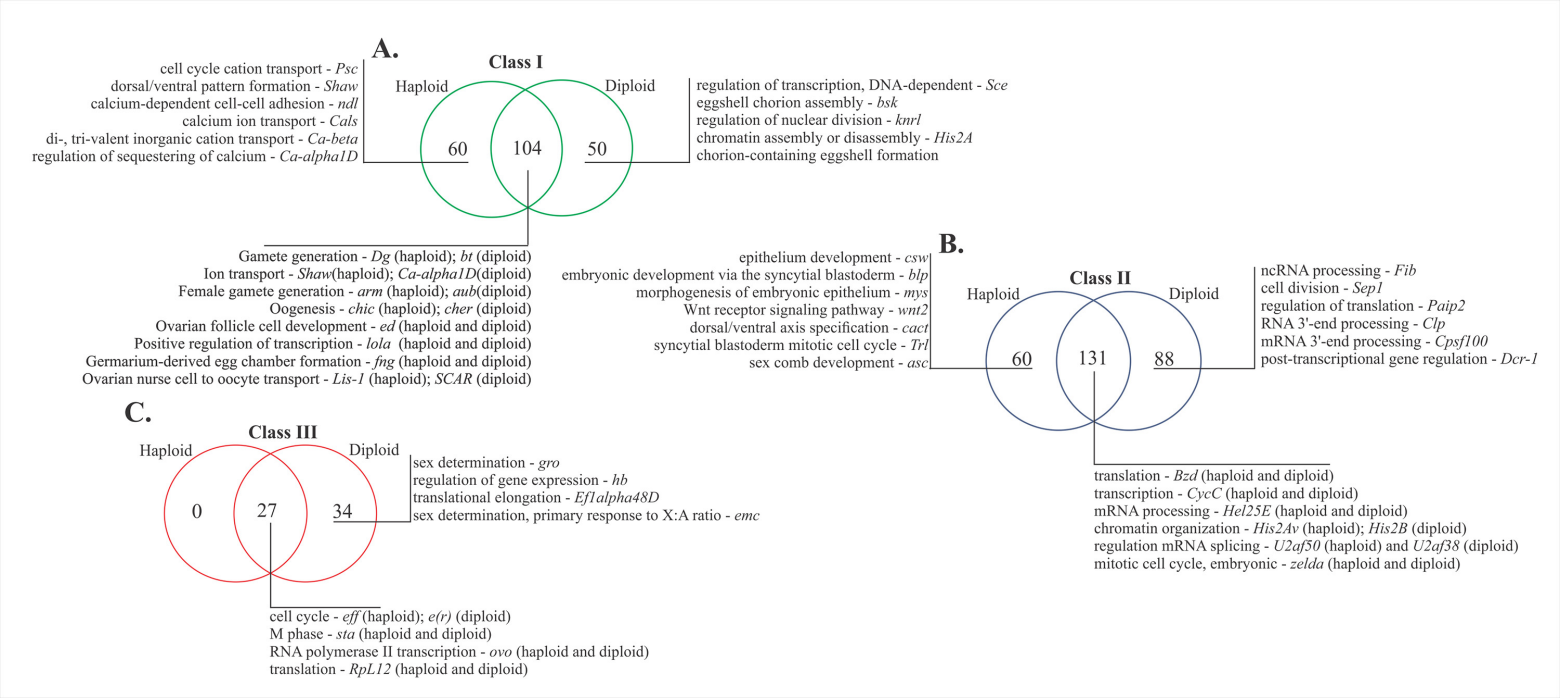
Gene Ontology analysis. **The main biological processes found for class I (A), class II (B) and class III (C) genes.** The exclusive biological processes and those shared between the haploid and diploid embryos in each class of mRNAs are highlighted according to their specificity in embryogenesis (for the complete GO terms, refer to S6 Table).

A GO analysis was performed to identify the biological processes to mRNAs of each class. The identified GO terms were compared to describe the similarities and differences between haploid and diploid embryos (Fig 4 and S6 Table). The terms “regulation of transcription” and “translation” were applied to the mRNAs of classes I, II and III; “oogenesis," “ovarian follicle cell development," “germarium-derived egg chamber formation," and “ovarian nurse cell to oocyte transport” associated to oogenesis processes were attributed to haploid and diploid class I mRNAs (Fig 4A). Interestingly, the biological process “calcium ion transport” was associated with the haploid class I, and it is an essential process for the first step in egg activation cascade (Fig 4A) [3,38]. Most of the class II mRNAs in both the haploid and diploid embryos were related to the “RNA processing” term. (Fig 4B). Processes related to blastoderm cellularization, such as “epithelium formation," “syncytial blastoderm” and “dorsal-ventral formation” were applied to the class II mRNAs of haploid embryos. In addition, class II genes, such as *zelda*/GB52047, were related to “regulation of transcription," “mitotic” and “cell cycle," which are processes clearly involved in the control of cell division. These processes corresponded to the most frequent events during cleavage (0–7 h) until blastoderm formation at approximately 24 h [20]. The transcripts of class III mRNAs in the haploid and diploid embryos were involved in the process of “cell division.” Interestingly, exclusive biological process terms did not apply to the mRNAs classified as class III in the haploid embryos (Fig 4C). The term “sex determination” however, applied to the class III mRNAs only in the diploid embryos.

### Putative TAGteam motifs in the early embryogenesis mRNA expressed of *A*. *mellifera*

**Fig 5:**
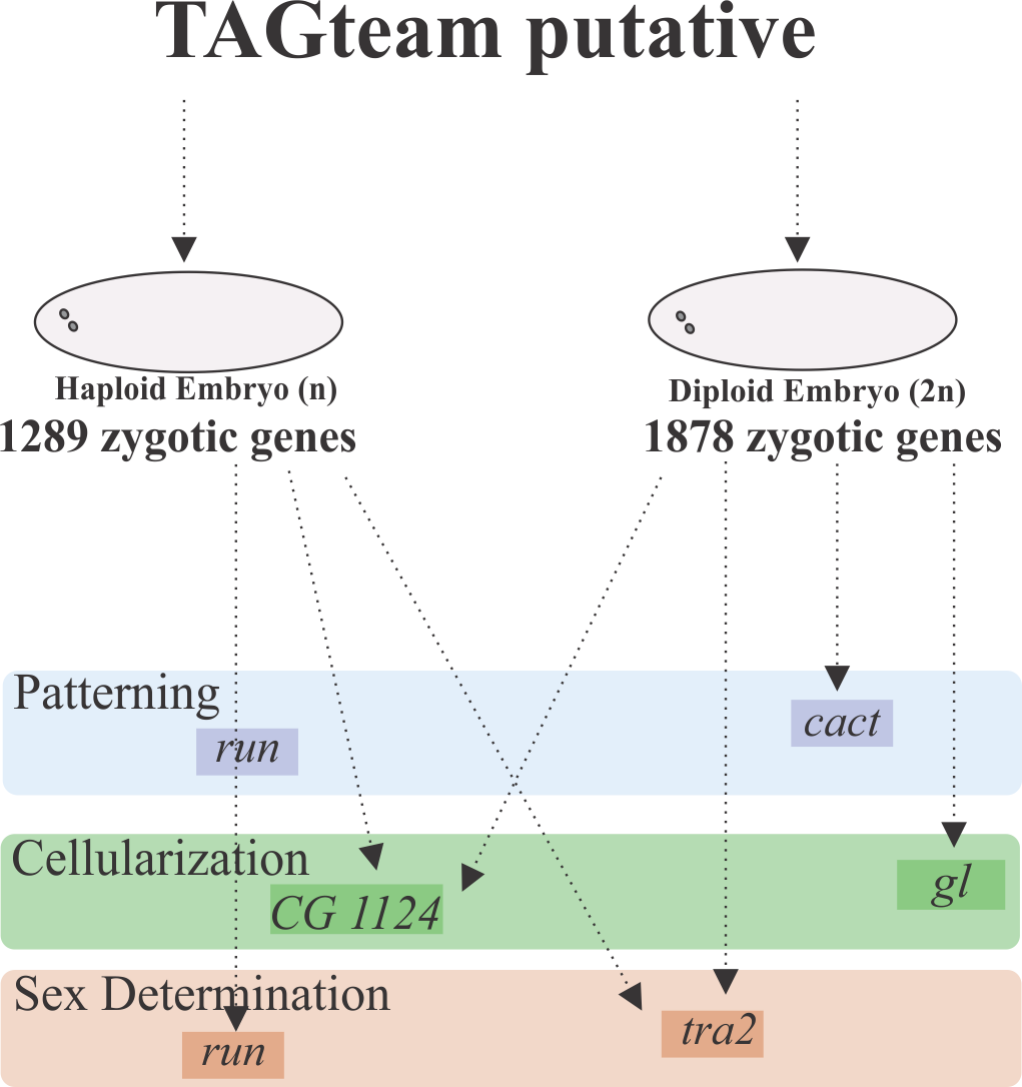
Diagram showing the number of mRNAs with the predicted TAGteam motif in the diploid and haploid mRNAs and examples of putative transcripts detected during the zygotic genome activation of *A*. *mellifera*. Putative TAGteam motif of *A*. *mellifera* localized in mRNAs with a zygotic expression profile (class II and class III) in the diploid and haploid embryos. Certain mRNAs were involved in patterning (*run* and *cact)*, cellularization (CG1124 and *gl*) and sex determination (*run* and *tra2*). The expression values of these mRNAs are in S3 Fig.

We searched for TAGteam motifs in the promoter region of honeybee class II genes. We used SCOPE to search for the candidate motifs in the promoter region of mRNAs class II in the haploid and diploid embryos. We chose this class because of the dynamics of their expression, mRNAs whose expression profiles were characterized by an increased number of reads from cleavage to blastoderm. The analysis recognized a candidate AGGTA-containing motif in the haploid and diploid embryos (S1 File and S7 Table). We aligned the candidate with TAGteam motifs found in *Drosophila* [13,14] and *A*. *aegypti* [18] (Table 2). The motif AGGTA showed good alignment (*pvalue*) and was selected as the putative TAGteam motif of *A*. *mellifera*. In addition, TAGteam motifs were found in promoter region of honeybee mRNAs involved in “patterning” processes (*run* in the haploid and *cact/cactus*/GB53301 in the diploid embryos), “cellularization” (CG1124/GB42702 in the diploid and haploid, and *gl/glass*/GB43258 in the diploid embryos) and “sex determination” (*run* in the haploid and *tra2/transformer 2*/GB47305 in both types of embryos, haploid and diploid [13,15,16] (Fig 5 and S3 Fig).

**Table 2:** TAGteam motifs aligned with TAGteam candidate selected for *A*. *mellifera*. Comparison of the selected *A*. *mellifera* motif with the *Drosophila* and *Aedes* TAGteam motifs using STAMP. The same e-values were observed in the majority of alignments.

Identification	Organism	From	e-value	alignments
PWM* of TAGteam from Renzis *at al*.	*Drosophila*	Biedler, et al. 2012	1.19E-07	TACCT_ TACCTG
PWM* of TAGteam from ten Bosch *et al*.	*Drosophila*	Biedler, et al. 2012	7.02E-07	_TACCT_ CTACCTG
Sequence of TAGteam1	*Drosophila*	ten Boch, et al. 2006	7.02E-07	_TACCT_ CTACCTA
Sequence of TAGteam2	*Drosophila*	ten Boch, et al. 2006	7.02E-07	_TACCT_ CTACCTG
>EZG_VBRGGTA_400	*Aedes*	Biedler, et al. 2012	3.71E-06	TACCT_ _ TACCTRY

*PWM: Position weight matrix

**Fig 6:**
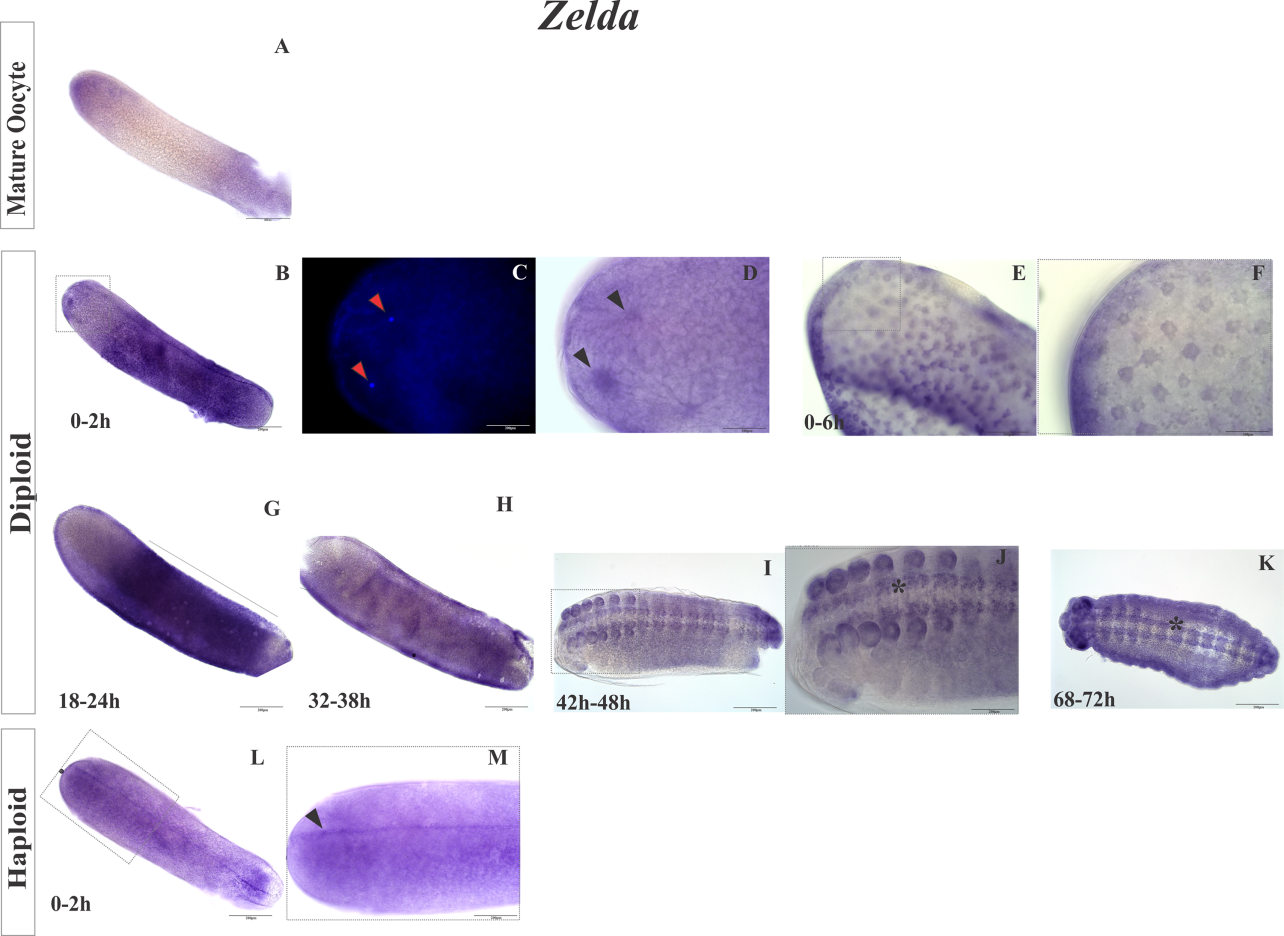
Location of *zelda* expression in the embryos of *A*. *mellifera* according to *in situ* hybridization. (A) *Zelda* expression in a mature oocyte with stronger staining at the anterior pole. (B, C, and D) Embryo within 2 h of development. (C and D in detail) Close to dividing nuclei positive reaction indicating foci of *zelda*. (E and F) Embryo with 6 h of development showing (in detail) the expression of *zelda* in the cleavage nuclei at the anterior pole. (G) *zelda* detected in 18–24 h embryos during the blastoderm stage. The gene product was concentrated in the central region of the embryo. (H) *zelda* detected in the blastodermic cells in 32–38 h embryos at the beginning of gastrulation. (I and J) Embryo at 42–48 h and (K) 68–72 h of development showing *zelda* expression in the primordial cells of the nervous system. Asterisk (*) indicates the ventral nerve cord in J and K. (L and M) Haploid embryos at 0–2 h of development showing *zelda* as an anterior-posterior stained strip along the embryo (Arrow in M).(A, B, G, M, L, and E): 200 μm scale bar, objective 10X; (E, J, and M): 100 μm scale bar, objective 20X on an Olympus BX61 microscope; (C, D, and F): 50 μm scale bar, 40X objective; (C): DAPI staining. The negative controls are in S4 Fig.

We can speculate that Zelda is a similar transcriptional activator of the zygotic genome in honeybees as in *Drosophila* [13,15]. Expression of *zelda* was detected at the beginning of honeybee embryogenesis (Fig 3), and we used *in situ* hybridization to localize the transcripts of *zelda* in the honeybee embryos. Transcripts of *zelda* were found at the anterior pole of mature oocytes (Fig 6A) near the first cleavage nuclei (0–2 h and 0–6 h, Fig 6B and 6D–6F) and in blastoderm cells (18–24 h, Fig 6G) as well as at the beginning of gastrulation (32–38 h; Fig 6H), during morphogenesis in ectodermal cells (42–48 h; Fig 6I), and in the precursor cells of the central nervous system (CNS) in late diploid embryos (68–72 h; Fig 6J and 6K). In 0–2 h haploid embryos, *zelda* mRNA was found to be dispersed in an anterior-posterior line (Fig 6L and 6M, arrow). Transcripts for Zelda were found in mature oocytes; however, it is not possible to know whether *zelda* transcripts were transcribed by follicular cells and deposited in the developing oocyte or by the developing oocyte itself. Besides being expressed in the early embryogenesis, *zelda* was also expressed during late embryonic development (Fig 6J and 6K, asterisk). We observed a concentration of *zelda* mRNA in the CNS precursors cells, including in the commissures and cephalic region in the 68–72 h embryos (Fig 6K) as described for *Drosophila*, where Zelda is involved in CNS formation [56].

### Predicted miRNA-target interactions as a model for differential regulation in early haploid and diploid embryos

**Fig 7:**
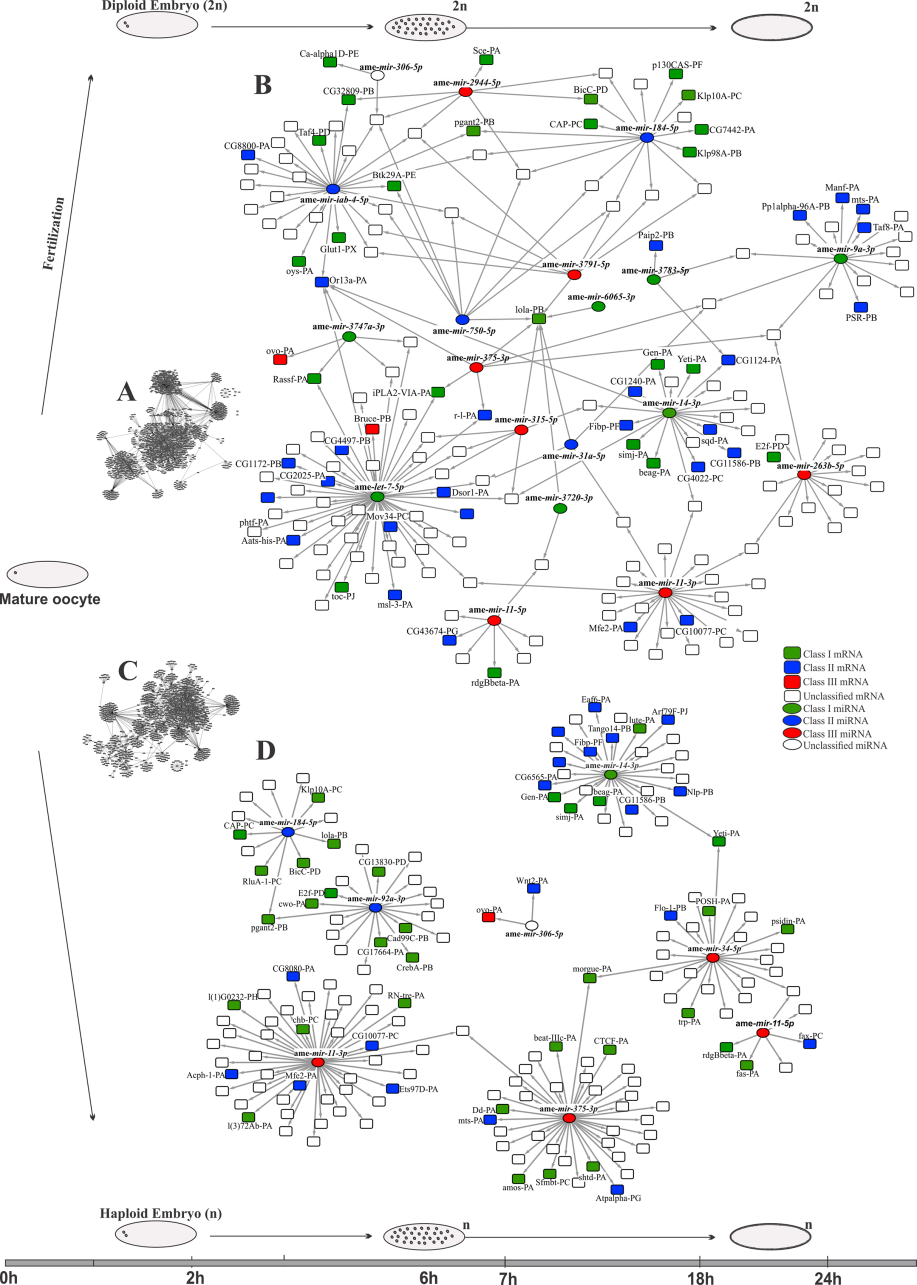
Predicted miRNA-target interactions for the diploid (A and B) and haploid (C and D) embryos of *Apis mellifera* during the early steps of embryogenesis. (A and C) Complete network and (B and D) reduced networks showing the predicted miRNA-target interactions and focusing on specific genes. Different colors and shapes identify class I, class II and class III miRNAs and genes. Note that the networks denote specific interactions for haploid and diploid embryos.

Based on the predicted interactions between miRNA and targets and in the expression profiles of mRNAs and miRNAs (S8 Table), we reconstructed networks comparing the putative role of miRNAs in early embryogenesis of honeybee haploid (Fig 7A and 7B) and diploid embryos (Fig 7C and 7D).

In diploid embryos (Fig 7A and 7B), a higher number of miRNA-target interactions (2,044) were predicted compared to haploid embryos (1,678) (Fig 7C and 7D). Ame-*miR-34-5p* presented putative targets only in haploid embryos (Fig 7), despite both ame-*miR-34-5p* and ame-*miR-34-3p* being expressed in both types of embryos (Fig 3). Ame-*miR-375-5p*, classified as class III in haploid embryos and as class II in diploid embryos, has more predicted targets in haploid than in diploid embryos. Other miRNAs also presented different profiles and targets when diploid and haploid embryos were compared. As examples, we cite ame-*miR-263b-5p* (class I in haploid and class III in diploid embryos), ame-*let-7-5p* (class III in haploid and class I in diploid embryos), and ame-*miR-iab-4-5p* (a class II miR present only in diploid embryos), but with predicted targets in only one type of embryo (ame-*miR-263b-5p* only in haploid, ame-*let-7-5p* and ame-*miR-iab-4-5p* in diploid) (see Fig 7B and 7D). Another miRNA with many targets in both types of embryos was ame-*miR-11-3p*, a class III gene (Fig 3), and ame-*miR-11-5p*, although this occurred with fewer mRNA-target interactions relative to ame-*miR-11*-3p (Fig 7).

### Primary miRNA transcripts detected in early embryos reveal zygotic transcription during cleavage

**Fig 8:**
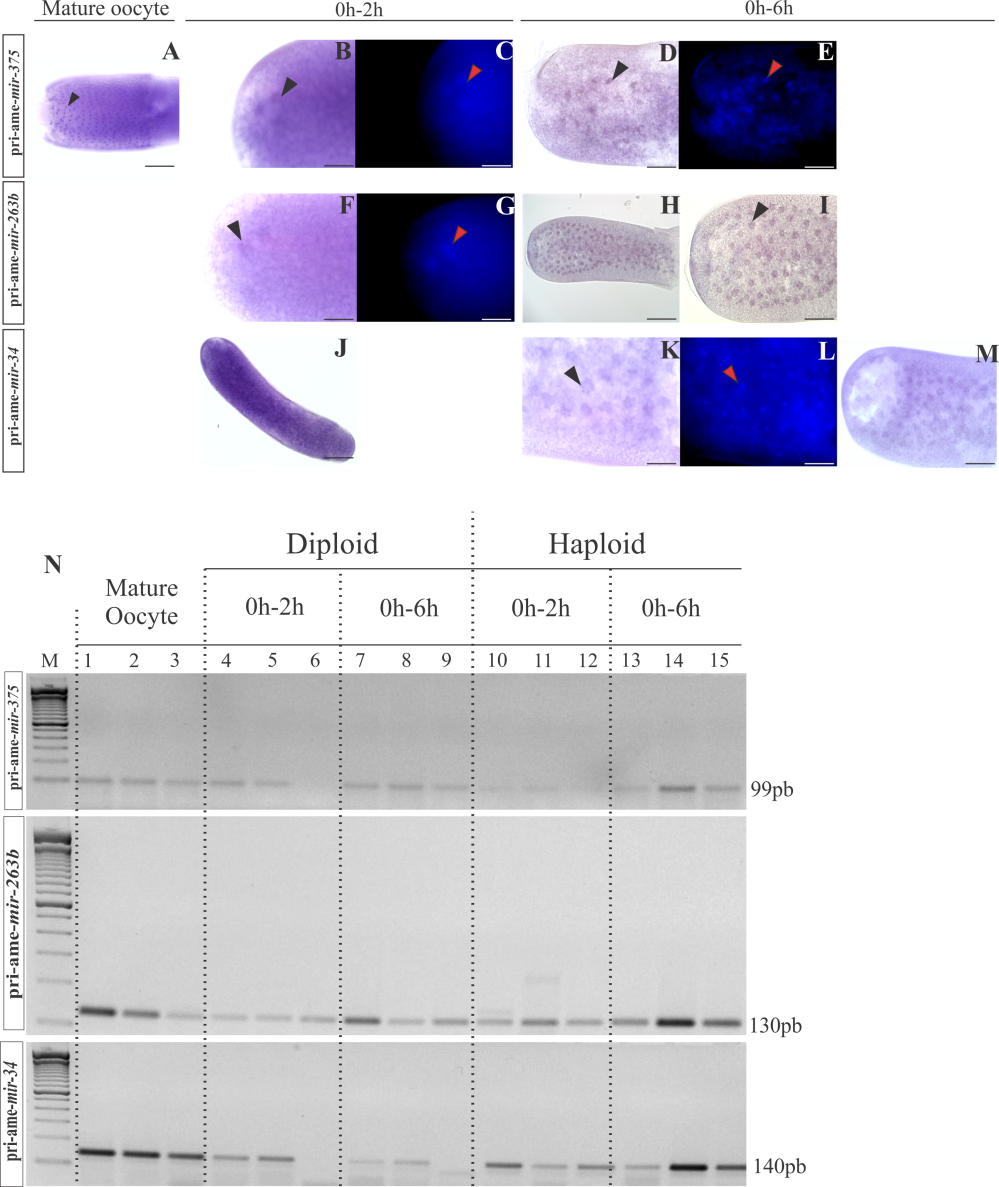
Expression of pri-ame-*mir-375*, pri-ame-*mir-263b*, and pri-ame-*mir-34* in the oocytes and during early embryonic development of *Apis mellifera*. *In situ* hybridization (A-B, D, F, H-K, and M) and PCR assay (N) showing a positive reaction for all tested mRNAs in the early phases of development, thus indicating the synthesis of miRNAs. In the embryos, the reaction was localized around the nuclei, and the PCR assay revealed the expression of pri-miRNAs in all of the analyzed phases. In the PCR assay, U6 was used as a positive control (additional qPCR experiments confirmed these results; data not shown). The presence of nuclei in the early embryo was determined using DAPI staining (C, E, G, and L). (A, D, E, I and M) 200 μm scale bar and 10X objective; (B, C, F, G, K and L) 100 μm scale bar and 20X objective; (B, C, F, G, K and L) 50 μm scale bar and 40X objective on the Olympus BX61 microscope. The negative controls are in S4 Fig.

Several mRNAs and miRNAs increased their expression as the embryo developed from the oocyte to cleavage stages (0–2 h and 0–6 h) (Fig 3A and 3B), raising the possibility of the zygotic genome activation before the blastoderm stage in honeybees. To test this hypothesis, we assessed the expression profiles of three pri-miRNAs (ame-*mir-263b*, ame-*mir-34* and ame-*mir-375*) by *in situ* hybridization and RT-PCR. The *in situ* hybridization assay showed that the transcripts of the pri-miRNAs ame-*mir-263b*, ame-*mir-34* and ame-*mir-375* were located inside the nuclei in the early embryos of *A*. *mellifera* (Fig 8A–8M). Interestingly, we also found pri-ame-*mir-375* transcripts in the nuclei of follicle cells adhered to the eggs (Fig 8A). The transcripts of pri-ame-*mir-263b*, pri-ame-*mir-375* and pri-ame-*mir-34* were amplified by RT-PCR in the embryos at 0–2 h and 0–6 h (Fig 8N), thus confirming the presence of pri-miRNAs in the early embryogenesis of honeybees. Overall, our results suggest that the zygotic genome is already active during the cleavage stage in *A*. *mellifera*.

## Discussion

In this study, we analyzed 14 deep-sequenced mRNA and miRNA libraries from oocytes and age-controlled early embryos (haploid and diploid) of *A*. *mellifera*. In total, approximately 125 million reads were mapped to the *A*. *mellifera* database (Official Gene Set 3.2). The initial analysis confirmed the results from previous studies on honeybees. A number of important developmental mRNAs were missing, such as those involved in the early events of embryogenesis [22–31], although mRNAs related to cell signaling, such as *wnt*, *hh* and *notch*, were conserved [32]. However, not all of the mRNAs related to the early identification of germ cells or pole plasm were present in the honeybees [32], and other mRNAs may replace them, such as in the case of *bicoid* (*bcd)*, which is present in many insects but absent in honeybees. Authors have argued that its function is substituted by *orthodenticle* and *hunchback* [32] as shown in *Tribolium* [57].

### Honeybee early expression mRNAs and miRNA

The classification of miRNAs and mRNAs according to their expression profiles provided information on the MZT mRNAs in *A*. *mellifera*. This strategy has been used previously for other insects [17,19,32,58], and it has been shown to be a powerful tool for understanding important events of early development of holometabolous insects. The class I mRNAs presented an expression profile that suggested the degradation of maternally deposited mRNA, which was observed by Thomsen *et al*. [1]. The class II gene profiles showed increased gene expression, which may have been caused by zygotic transcription as reported by Benoit *et al*. [17] and Siddiqui *et al*. [19]. Similarly, the class III mRNAs suggested an expression profile characteristic of maternally deposited mRNAs that have been degraded during cleavage and then replaced during blastoderm formation by the zygotic machinery, which occurs in *Drosophila* and was reported by Benoit *et al*. [17]. Because several miRNAs and mRNAs presented a differential expression profile in the haploid and diploid embryos (Fig 3 and S5 Table), many of mRNAs were categorized differently in the sexes. For example, mRNAs found to be exclusive for one type of embryo, such as *wnt2* (class II, haploid), and mRNAs previously described as expressed in the embryogenesis of *A*. *mellifera* [32], such as *Dicer-1* (class II, diploid). However, for certain genes, similarities were observed in the classification of both types of embryos, such as the classification of *zelda* (class II), a fundamental gene involved in zygotic genome activation in *Drosophila* [13,15], *lola/longitudinals lacking* (class I), a gene involved in gonadal differentiation [59], and *ovo* (class III), a gene required for female germline differentiation [60]. The majority of the processes were shared between the two types of embryos. The class I genes, which are involved in processes linked to oogenesis, received the same classification in both types of embryos. The class II and III mRNAs responsible for the maintenance of cell division control, transcription, and translation received different classifications. These processes are recurrent in early embryogenesis until the blastoderm stage [20,36,61,62]. However, the peculiarities found in each type of embryo, such as sex determination (class III, diploid), calcium signaling (class I, haploid) and syncytial blastoderm (class II, haploid) indicated that the differentiation of males and females may occur during the initial development. Therefore, the basic processes of embryogenesis were maintained, although they lagged in time by at least one mitotic cycle according to Darbo *et al*. [63].

The difference in miRNA classification between the two types of embryos was more evident than in the mRNAs classification, which was most likely because of the independent expression of the 3p and 5p forms of the same miRNA. For example, ame-*let-7-3p* is a class II gene in both types of embryos and ame-*let-7-5p* is a class III gene in haploid embryos. We observed miRNAs with different arm use classified differently between the two types of embryos. Ame-*miR-375-3p* is class II gene in diploids and class III gene in haploids, and ame-*miR-263-5p* is a class I gene in haploids and class III gene in diploids. The main function of miRNA in the cell or organism is the regulation of target expression [64]. As described above, we observed that certain mRNAs were expressed differently during haploid and diploid development, and the same process occurred with the arms of certain miRNAs. This process is noticeable in the work of Jiang *et al*. [65], who observed antagonistic effects of miRNA arms on their targets and a consequent modification of cell behavior, including mobility. Regardless of the classification, most of the miRNAs presented high expression in the mature oocytes, which suggests that the mature miRNAs were maternally deposited. However, several miRNAs (ame-*miR-375-3p* in the diploid embryos and ame-*miR-184-3p* in the haploid embryos) showed a slight increase in the 0–2 h embryos (Fig 3B), suggesting that miRNAs could be processed during early embryogenesis if the precursor (pre-miRNA) was deposited during oogenesis, which may be possible because the mRNAs of the machinery for miRNA biogenesis are localized in both types of embryos (S2 Fig), an essential condition for the production of mature miRNAs.

### Putative TAGteam motif acts in honeybee early zygotic gene activation

In *Drosophila*, the protein Zelda binds to the TAGteam motif to activate target mRNAs transcribed in the first wave of zygotic transcription [13,15,16]. These mRNAs are mainly involved in sex determination, dorsal-ventral patterning, antero-posterior patterning, and cellularization [13]. Here, we found mRNAs with TAGteam motifs involved in sex determination (*run* and *tra2* in haploid embryos and *tra2* in diploid embryos), patterning (*run* in haploid embryos and *cact* in diploid embryos) and cellularization (*CG1124* in haploid embryos and *gl* in diploid embryos). These results showed that the system is conserved compared with that of *Drosophila*. In addition, this result was supported by the presence of miRNAs that are controlled by Zelda and function to maintain developmental timing [66]. In *Drosophila*, Zelda acts in the transcription of temporal miRNAs. Our analysis revealed that a proportion of these miRNAs are expressed during early embryogenesis, including ame-*iab-4-5p* in the diploid embryos, ame-*mir-92a-3p* in the haploid embryos, ame-*mir-9a-3p* in the diploid embryos, and ame-*mir-11-3p* and *5p* in both types of embryos (Fig 3), and its predicted function is the degradation of maternal genes, *Hox* gene regulation, and chromatin accessibility [66]. Nevertheless, in honeybees, the analysis also revealed differences between the diploid and haploid embryos. For instance, *run* is a gene involved in sex determination, and it was only detected in diploids. This result indicates that sex determination in each type of embryo is dynamically different [33,67]. Our results show that the MZT occurs similarly in haploid and diploid embryos at least until 24 h of development during blastoderm cellularization. In *Drosophila*, this event occurs during the same phase but at a different time because its embryogenesis is shorter than in honeybees [2,6,17].

The gene *zelda* encodes a DNA binding protein and is a transcription factor that is considered to be the major regulator of zygotic genome activation in *Drosophila* [13,15,68]. The presence of *zelda* in our libraries further suggested that its corresponding binding motif, AGGTA (TAGteam putative), plays a role in *A*. *mellifera* development as well. Moreover, *in situ* hybridization suggested that the function of *zelda* is conserved in *A*. *mellifera*. The presence of *zelda* mRNA around the nuclei in early embryos (Fig 6) implies that it is maternally deposited despite being classified as zygotic (Fig 3). In *Drosophila*, Zelda is a nuclear protein that is detected 1–2 h into embryonic development [69]. *Zelda* mRNA was also stained in blastodermic cells, precursors of the CNS, the ventral nerve cord and the brain in honeybees. Other ectodermal derivatives were also stained, including the stomodeo cells and mouth-part precursors, and their roles in early embryogenesis, zygotic gene transcription during the blastodermic stage [15], late embryogenesis, neuronal tissue precursors and the tracheal system have been described in *Drosophila* by Pearson *et al*. [56].

### Differentially regulated mRNAs in the early haploid and diploid embryos by miRNAs

Predicting the interactions between a regulator (miRNA or transcription factor) and its target is an approach to exploring gene functions in a system or developmental period. For the miRNA-target searches, we used antagonistic expression data [70,71] and the interaction between miRNA and the 3’UTR of the candidate mRNAs [52,53]. In this study, we show that miRNAs differentially regulate mRNAs at the beginning of embryonic development in *A*. *mellifera* in the haploid and diploid embryos. The spatial and temporal co-expression of miRNA and its target mRNAs and the predicted binding sites for miRNA on the 3’UTR of the target gene [64] strongly indicate miRNA-target interactions and regulation. The decreased target-mRNAs levels caused by a probable miRNA action, which was revealed by the antagonistic profiles, reinforce the evidence of a miRNA-target interaction as described by Giraldez *et al*. [72], Mishima *et al*. [73] and Bethune *et al*. [74]. In *Drosophila*, Zelda regulates *mir-9a*, *mir-92a*, *mir-11* and *iab-4* [66]. However, even though Zelda could regulates the same mRNAs in honeybees, our analyses indicate differences between both types of embryos, with *ame-iab-4-5p* identified only in diploid embryos. The mRNAs in *Drosophila* described as being involved in maternal mRNA degradation during the MZT and regulated by a specific miRNA [17] were also found in our analysis; for example, *smg-PE* (*smaug*/GB48452) was regulated by *ame-miR-11-5p*, suggesting that the MZT is a conserved process. The interactions between regulators and targets appear to be specific for diploid and haploid embryos, especially in the analyses of the expression profiles during development. The highly conserved gene among metazoans, *let-7* [66–68], is a good example. In honeybees, this miRNA also has many possible targets during early embryogenesis, although there are marked differences between the diploid and haploid embryos. The expression of ame-*miR-34* reinforces this type of difference observed between both types of embryos. In *Drosophila* and zebrafish, ame-*miR-34* is maternally deposited [8], which is similar to honeybees. However, despite the suggestion of a conserved function, at least during oogenesis, this miRNA behaves differently at the beginning of development in the diploid and haploid embryos. The predicted interactions suggested different targets for ame-*miR-34* during this period of development for both types of embryos, and this also occurred for ame-*miR-263b-5p* and ame-*miR-375-3p*, although during different periods of development. The detection of such variations implies transcription by zygotic machinery, a hypothesis that we tested using probes designed for the detection and localization of pri-miRNA in *in situ* hybridizations experiments of three miRNA (ame-*mir-34*, ame-*mir-263b* and ame-*mir-375*).

### Honeybee zygotic genome activation occurs during cleavage

Determining the localization of pri-miRNA during development provides support for its classification and origin. Along with a PCR assay, this approach also revealed zygotic transcription during the early cleavage stages in honeybees. For many years, zygotic transcription was considered to be repressed during cleavage [68]. However, in the nematode *Ascaris suum*, the majority of zygotic transcription occurs between fertilization and fusion of the pronuclei, implying that zygotic mRNAs and the deposited maternal products drive early embryogenesis in *A*. *suum* [75]. The presence of essential mRNAs for miRNA biosynthesis in our libraries of early embryos (S2 Fig) indicated that the newly produced miRNAs could be processed. In addition, mRNAs were observed with a zygotic profile (class II) that increased their expression from mature oocyte to 0–2 h and 0–6 h embryos, which allowed us to conclude that in *A*. *mellifera*, the zygotic genome is activated during early cleavage and new molecules are produced.

## Supporting Information

S1 Table Expression of mRNAs (mRNAs) and miRNAs in the early embryogenesis of *A*. *mellifera*. (XLSX)

S2 Table BLAST search of the orthologous mRNAs in *Drosophila*. (XLSX)

S3 Table DEGseq output. Differentially expressed mRNAs.
(XLSX)

S4 Table DEGseq output. Differentially expressed miRNAs.
(XLSX)

S5 Table Classification of mRNAs and miRNAs based on their expression profile (DEGseq) in the early embryogenesis of *A*. *mellifera*. And Expression (RPKM) and DEGseq output of haploid and diploid embryos highlights mRNAs and miRNAs in the Fig 3.
(XLSX)

S6 Table Gene Ontology analysis (Biological Processes). DAVID output.
(XLSX)

S7 Table mRNAs that contain the selected motif ATCAA, TAGteam putative of *A*. *mellifera*. (XLSX)

S8 Table Predicted miRNA-target information. (XLSX)

S1 Fig Length of the mapped reads of the small RNA libraries. (TIF)

S2 Fig Expression of machinery for miRNA biogenesis genes. (TIF)

S3 Fig Expression profile of TAGteam putative genes of Fig 5. (TIF)

S4 Fig *In situ* hybridization negative controls. (TIF)

S1 File Information on the motif search. (PDF)
