# Supplementary figures and images for: Transcriptome Analysis of Honeybee (Apis Mellifera) Haploid and Diploid Embryos Reveals Early Zygotic Transcription during Cleavage
Source: PLoS One. 2016 Jan 11;11(1):e0146447. doi: 10.1371/journal.pone.0146447 (PMC4713447; doi:10.1371/journal.pone.0146447)

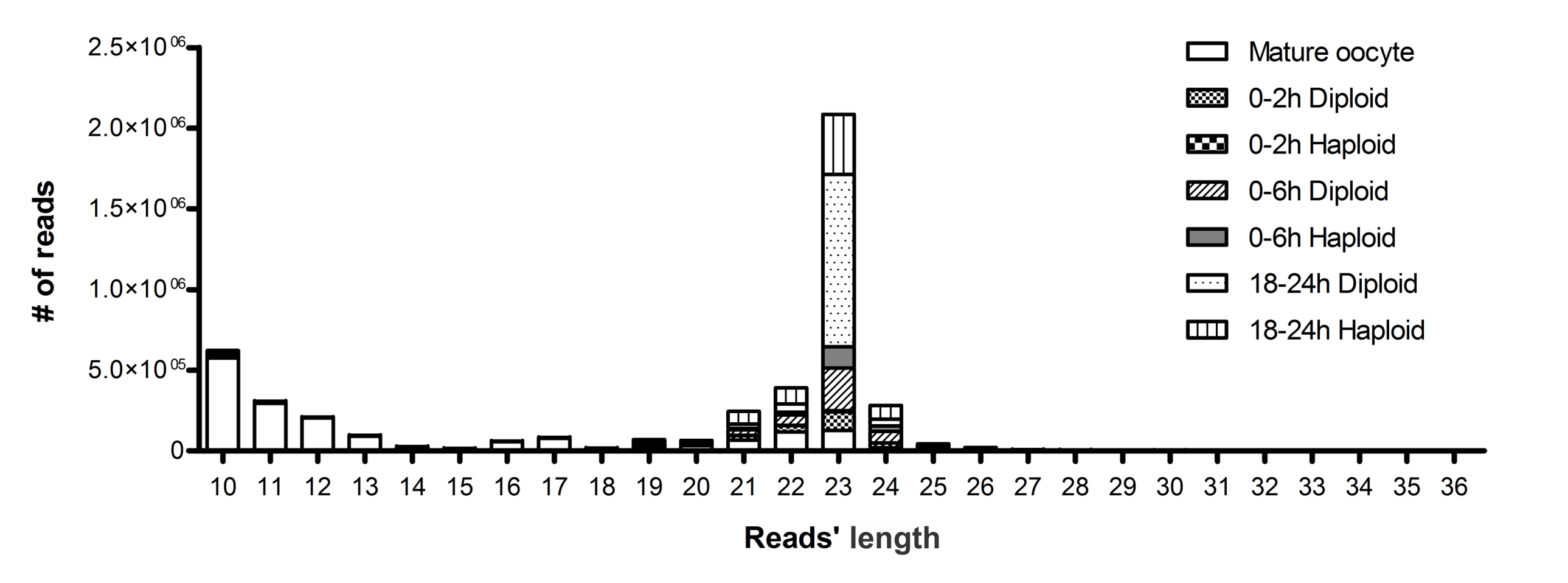

Supplement: S1 Fig — (TIF) [file pone.0146447.s009.tif]

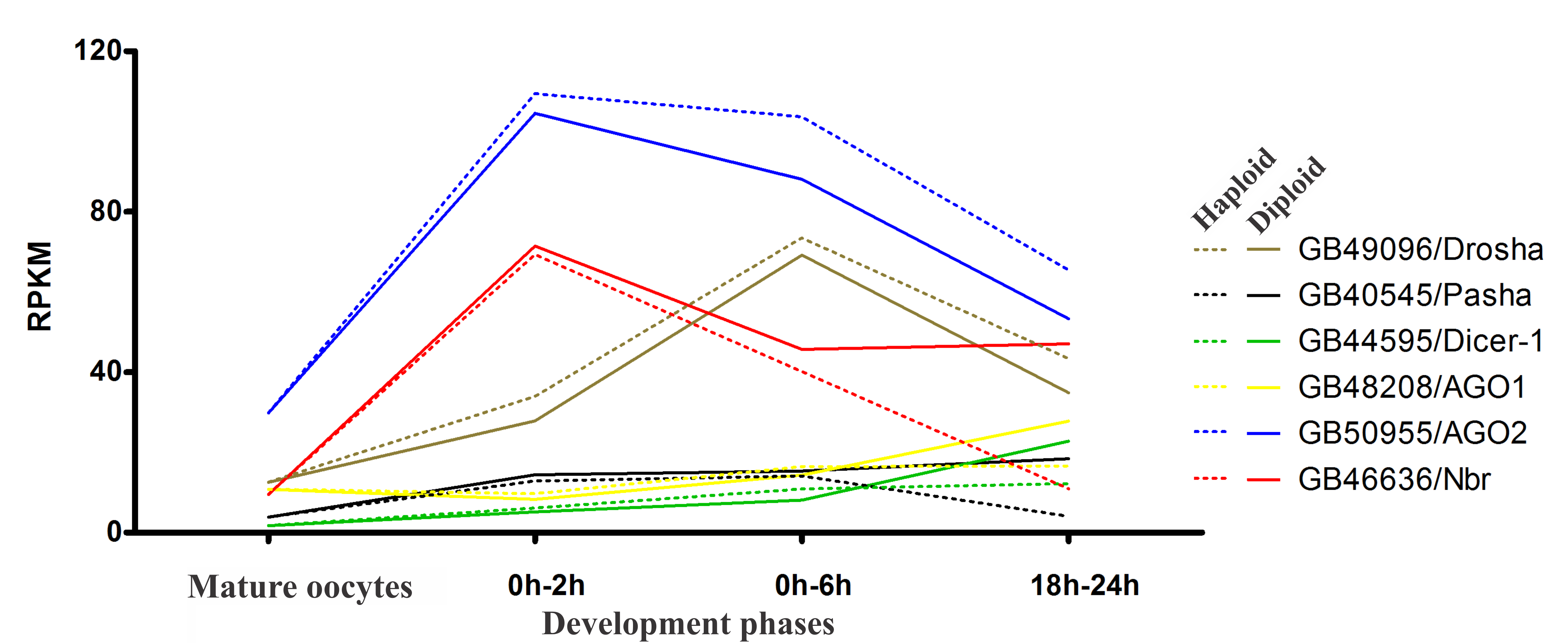

Supplement: S2 Fig — (TIF) [file pone.0146447.s010.tif]

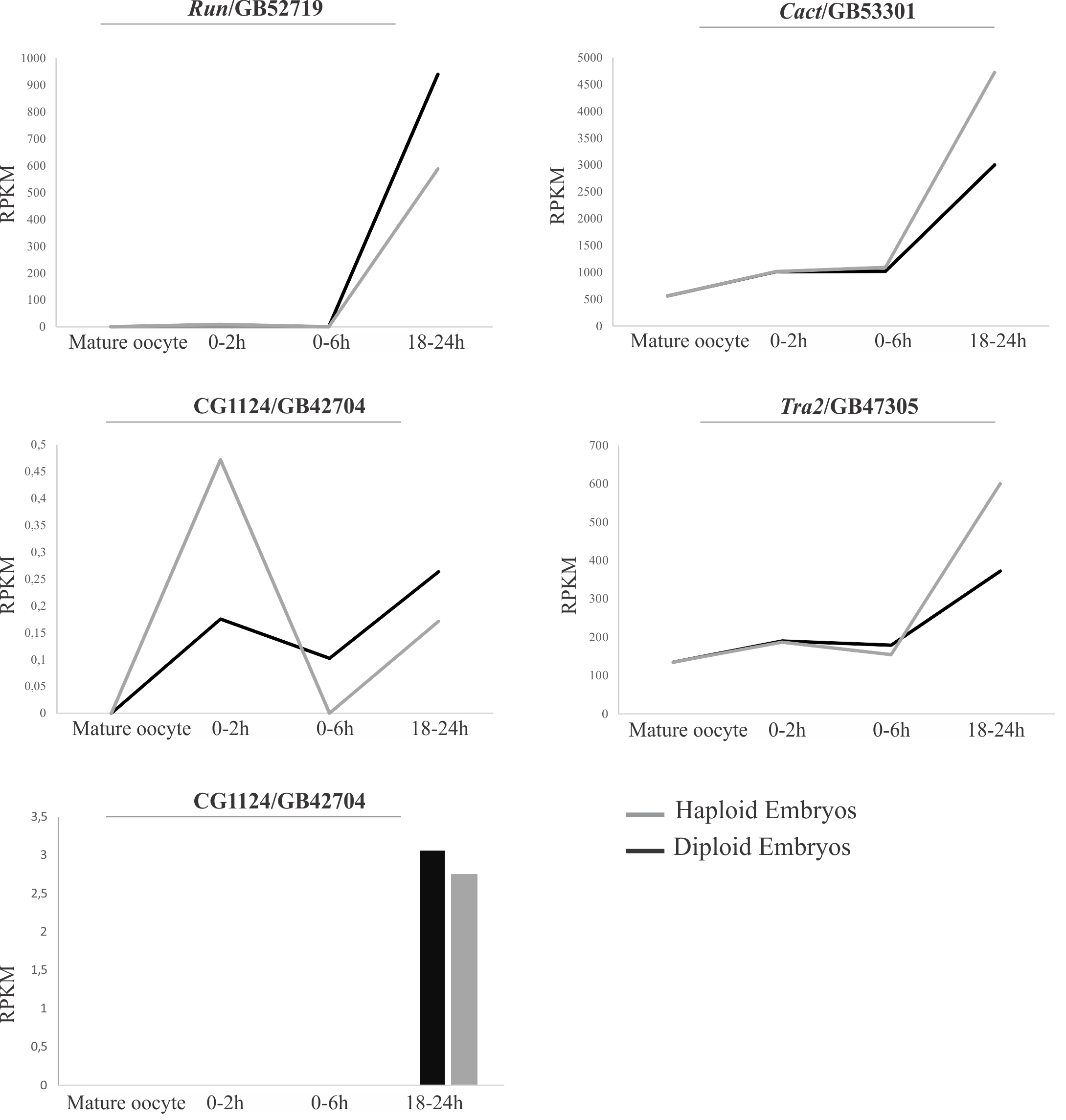

Supplement: S3 Fig — (TIF) [file pone.0146447.s011.tif]

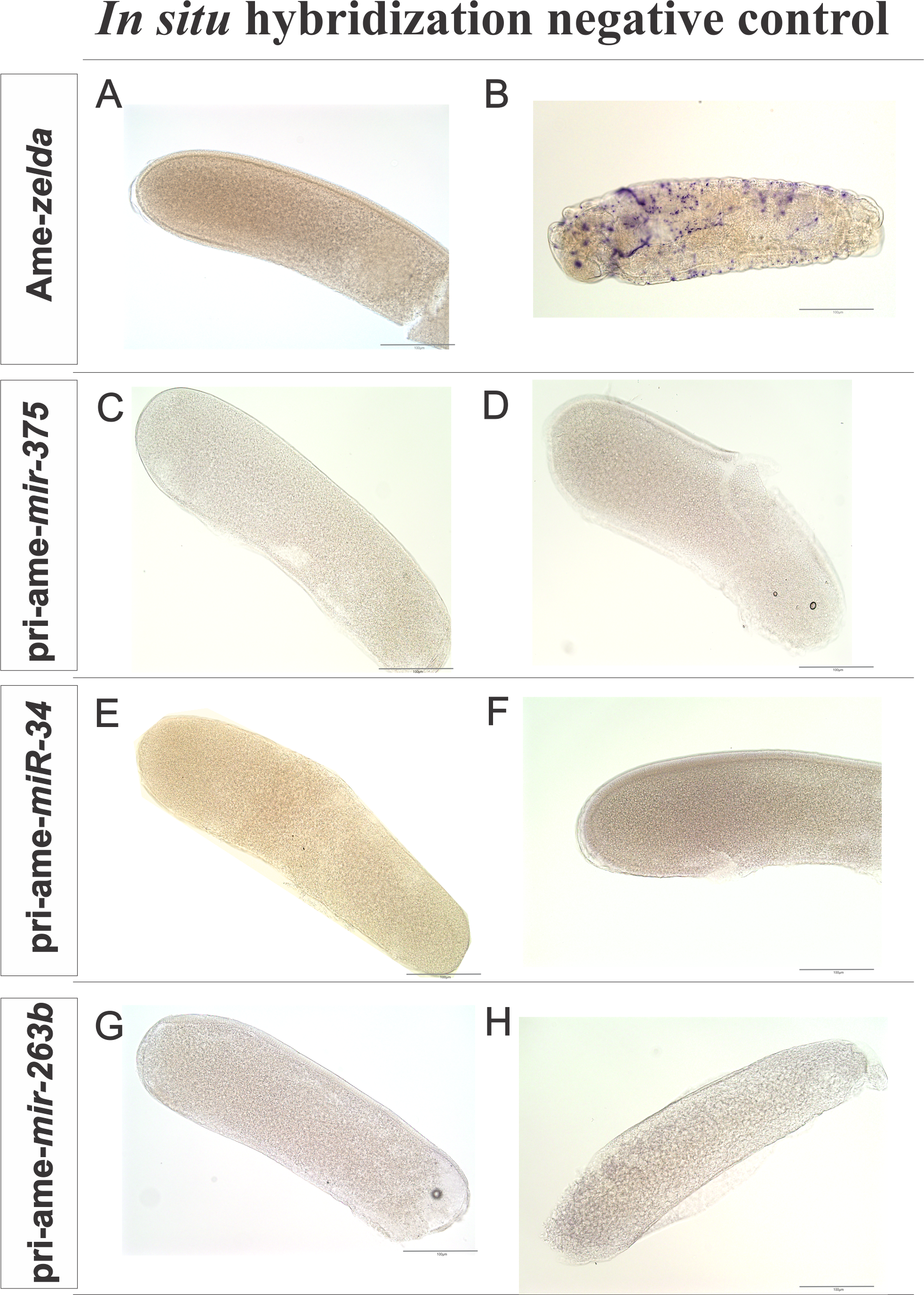

Supplement: S4 Fig — (TIF) [file pone.0146447.s012.tif]
