# Supplementary material for: Transcriptome Analysis of Honeybee (Apis Mellifera) Haploid and Diploid Embryos Reveals Early Zygotic Transcription during Cleavage
Source: PLoS One. 2016 Jan 11;11(1):e0146447. doi: 10.1371/journal.pone.0146447 (PMC4713447; doi:10.1371/journal.pone.0146447)
Supplement: S1 File — (PDF) [file pone.0146447.s013.pdf]

S8: Information of motif search.

1: SCOPE output of consensus sequence of TAGteam putative of *A. mellifera* found in the promoter of zygotic genes class, haploid embryos (A) and diploid embryos (B). The selected motif is in purple.

(A)

	Consensus Sequence	Count	Sig Value	Coverage
#1	bnccad	17402	545.4416	96.10%
#2	aadatw	60960	250.0416	97.10%
#3	hagndgha	7242	217.9103	87.00%
#4	ctnndgcw	2589	201.103	56.30%
#5	aaata	37239	182.7164	96.90%
#6	atgga	4393	148.9229	77.80%
#7	ccaaa	3453	128.2851	72.00%
#8	aaatt	35892	127.7127	96.50%
#9	ctgca	1867	122.3791	48.60%
#10	ctgga	1694	112.7276	46.80%
#11	atcca	3756	109.4426	72.30%
#12	agaag	5500	102.4758	78.20%
#13	**aggta**	2205	78.6195	56.60%
#14	ascwddwrcag	252	76.3445	4.20%
#15	ctgaa	3365	75.8061	72.80%
#16	agcnnnagc	476	73.0569	10.80%
#17	cagnngca	568	71.9061	15.10%
#18	gatga	4491	71.3759	77.80%
#19	ccaag	1551	68.5935	42.20%
#20	aaacc	2433	61.3895	59.60%
#21	agcnnaag	644	61.344	18.20%
#22	agctc	1113	61.1016	32.60%
#23	atggg	1146	60.7781	35.80%
#24	accat	2509	59.8637	61.90%
#25	cagnnncag	470	59.5073	12.00%
#26	accag	1300	58.2172	38.60%
#27	acatc	3211	55.2039	71.60%
#28	atcag	2792	54.4725	65.90%
#29	aggat	3160	54.3256	67.40%
#30	ctgnnnntgc	486	51.6271	13.10%

(B)

	Consensus Sequence	Count	Sig Value	Coverage
#1	cagnwncw	4516	396.2405	0.665
#2	cagnwnha	9937	353.5446	0.907
#3	scwnhwgcw	3087	345.3769	0.456
#4	akgbah	34222	311.3976	0.967
#5	aattnt	32331	250.097	0.955
#6	ttaaa	25869	246.4297	0.946
#7	ttaat	24329	225.1557	0.95
#8	ccaaa	5964	178.2223	0.831
#9	**atgaa**	20215	173.3149	0.958
#10	tatca	15412	167.0161	0.94
#11	accat	4486	166.5601	0.742
#12	aacaa	18100	161.065	0.948
#13	ctaaa	10338	156.1421	0.912
#14	accag	2471	153.0067	0.515
#15	aggta	4017	151.874	0.704
#16	ascwdnwncag	711	139.2318	0.143
#17	agcag	2993	134.1849	0.549
#18	atgga	7340	133.5298	0.862
#19	ctgga	2872	132.0334	0.576
#20	ctgca	3142	130.6922	0.596
#21	aagct	4241	129.4887	0.665
#22	caaga	6819	125.8912	0.856
#23	tacaa	15943	124.6433	0.939
#24	aagaa	25856	116.7011	0.958
#25	taaca	12174	111.2674	0.921
#26	caaca	6632	104.379	0.835
#27	agcnnnagc	810	103.8675	0.158
#28	ctata	10319	101.5278	0.894
#29	agcat	4954	98.7163	0.782
#30	ctgaa	5705	98.6594	0.821

2: PWM (position weight matrix) of selected motif as TAGteam putative of *A. mellifera* **(ATGAA).**

PWM:					
a	4017	0	0	0	4017
c	0	0	0	0	0
g	0	4017	4017	0	0
t	0	0	0	4017	0

3: PWM (position weight matrix) used for alignment in STAMP. This PWM was obtained in Biedler, *et al* (2012)

>EZG_VBRGGTA_400 (PWM from *Aedes aegypt* found by Biedler *et al* 2012)

A	C	G	T
47	20	37	0
0	29	16	59
64	0	40	0
0	0	104	0
0	0	104	0
0	0	0	104
104	0	0	0

>YAGGTA_PWM_Renzis_400 (PWM from *Drosophila* found by De Renzis *et al* 2007)

A	C	G	T
0	100	0	43
143	0	0	0
0	0	143	0
0	0	143	0
0	0	0	143
143	0	0	0

>YAGGTAG_PWM_Bosch_2500 (PWM from *Drosophila* found by ten Bosch *et al* 2006)

A	C	G	T
0	45	0	20
65	0	0	0
0	0	65	0
0	0	65	0
0	0	0	65
65	0	0	0
0	0	65	0

Sequences of TAGteam motifs from Drosophila, obtained in ten Bosch *et al (*2006)

>TAGteam1
TAGGTAG
>TAGteam2
CAGGTAG
>TAGteam3
CAGGCAG
